# Predator size affects the intensity of mutual interference in a predatory mirid

**DOI:** 10.1002/ece3.7137

**Published:** 2020-12-29

**Authors:** Nikos E. Papanikolaou, Sofia Dervisoglou, Argyro Fantinou, Theodore Kypraios, Valmari Giakoumaki, Dionysios Perdikis

**Affiliations:** ^1^ Laboratory of Agricultural Zoology and Entomology Department of Crop Science Agricultural University of Athens Athens Greece; ^2^ Department of Plant Protection Products and Biocides Hellenic Ministry of Rural Development and Food Athens Greece; ^3^ Laboratory of Ecology and Environmental Science Department of Crop Science Agricultural University of Athens Athens Greece; ^4^ School of Mathematical Sciences University Park University of Nottingham Nottingham UK

**Keywords:** competition, Crowley–Martin model, functional response, *Macrolophus pygmaeus*, mutual interference, omnivorous predator

## Abstract

1. Interference competition occurs when access to an available resource is negatively affected by interactions with other individuals, where mutual interference involves individuals of the same species. The interactive phenomena among individuals may be size‐dependent, since body size is a major factor that may alter prey consumption rates and ultimately the dynamics and structure of food webs.
2. A study was initiated in order to evaluate the effect of mutual interference in the prey‐specific attack rates and handling times of same size class predators, incorporating variation in consumer size. For this purpose, laboratory functional response experiments were conducted using same age predators, that is, newly hatched (first instar) or mature (fifth instar) nymphs of the polyphagous mirid predator *Macrolophus pygmaeus* preying on *Ephestia kuehniella* (Lepidoptera: Pyralidae) eggs.
3. The experiments involved four predator density treatments, that is, one, two, three, or four predators of same age, that is, either first‐ or fifth‐instar nymphs, which were exposed to several prey densities. The Crowley–Martin model, which allows for interference competition between foraging predators, was used to fit the data.
4. The results showed that mutual interference between predator's nymphs may occur that affect their foraging efficiency. The values of the attack rate coefficient were dependent on the predator density and for the first‐instar nymphs were significantly lower at the highest predator density than the lower predator densities, whereas for the fifth‐instar nymphs in all density treatments were significantly lower to that of the individual foragers' ones.
5. These results indicate that mutual interference is more intense for larger predators and is more obvious at low prey densities where the competition level is higher. The wider use of predator‐dependent functional response models will help toward a mechanistic understanding of intraspecific interactions and its consequences on the stability and structure of food webs.

## Graphical abstract

Mutual interference involves direct intraspecific interactions that may alter predator feeding behavior. A study was initiated in order to estimate the prey‐specific attack rates and handling times of a predatory mirid incorporating variation in the predator size. Our results indicate that mutual interference is more intense for larger predators and is more obvious only at low prey densities where the competition level is higher.

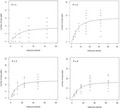

## 1 INTRODUCTION

Competition for resources is common in food webs (Kratina et al., 2009; Zhang et al., 2015), driving species distribution and evolution (Abrams, 2000; Craine & Dybzinski, 2013). Competitive interactions between individuals are assumed to be of two discrete types (Begon et al., 1996): exploitation competition, when species compete for the same limited resource, and interference competition in which one individual actively interferes with another individual's access to a resource. Both types of competition have negative effects mainly on the fitness of the weaker competitors (Lang & Bendow, 2013; McCormick & Weaver, 2012). Furthermore, mutual interference involves direct interactions between individuals of the same species that adversely may affect their searching efficiency, that is, the rate that a predator search for its prey, and ultimately its feeding rate (Beddington, 1975; DeLong & Vasseur, 2011; Hassell, 1978). Recently, Papanikolaou et al. (2016) showed that mutual interference may alter the feeding rate of aphidophagous coccinellid species, depending on predator density exposed in a patch.

Understanding the relationship between per capita predator consumption in increasing prey density, that is, the functional response (Solomon, 1949), is crucial for describing and estimating the feeding interactions between a consumer and a resource (see, e.g., Abrams, 1989; Englund et al., 2011; Jeschke et al., 2002; Sentis, Hemptinne, & Brodeur, 2012, 2013) and essentially the cornerstone for most quantitative ecological studies (Moffat et al., 2020; Zhang et al., 2018). Holling (1959a) proposed various types of functional response; in type I and II, the prey consumption is assumed to increase linearly with prey density or increase asymptotically, respectively, reaching a plateau, while in a type III functional response, the prey consumption is supposed of a sigmoid form as prey density increases. The well‐known disk equation, which describes type II functional responses, assumes that a single predator spends its time on two kinds of activities: prey searching and prey handling (Holling, 1959b); prey searching is depicted on the attack rate coefficient of the disk equation, representing the per capita prey eaten by a predator at low prey densities, that is, the densities that predator is not satiated, and determining the initial slope of the functional response curve; prey handling refers to the handling time coefficient, that is, the time a predator spends pursuing, subduing and eating a prey item, indicating the per capita maximum feeding rate at a given time. However, the disk equation is considered as a purely prey‐dependent functional response model because it did not incorporate parameters to assess behavioral interactions between foragers that may affect per capita functional responses due to competition. Nevertheless, intraspecific interactions act on predator search efficiency, and thus, predator dependence may facilitate predation or may reduce the potential of the interacting predators to locate and consume a prey. For example, Kratina et al., (2009) reported high encounter rates between individuals of the benthic flatworm *Stenostomum virginanum* when fed on *Paramericum aurelia* which reduced the available foraging time for the predator.

Extending Holling's assumptions, more realistic functional response modeling approaches have been developed to predict predation rates when the predator and the prey density vary. These models are considered as predator‐dependent (e.g., Beddington, 1975; Crowley & Martin, 1989; DeAngelis et al., 1975). Although the viability of these models has been shown (Skalski & Gilliam, 2001), their application remains underutilized in the ecological literature, and thus, our knowledge on how density dependence affects the linkages between predators and their prey resources remains little elaborated (e.g., Kratina et al., 2009; Papanikolaou et al., 2016).

Apart of predator density, the interactive phenomena among predators may be size‐dependent, since body size is a major factor that may alter prey consumption rates and ultimately the dynamics and structure of food webs (Christensen, 1996; Fuiman, 1989; Gravel et al., 2013; Mittlebach, 1981; Tsai et al., 2016; Yodzis & Innes, 1991). Similarly, body size may alter predator foraging capacity and may lead to changes in competitive ability and predator–prey dynamics (Hin & de Roos, 2018; Yodzis & Innes, 1991). For example, Thorp et al. (2018) reported that predator body size alters the type of functional response of the African clawed frog, *Xenopus laevis* (Anura: Pipidae), to *Culex pipiens* (Diptera: Culicidae); small predators exhibited a type II response, while medium and large predators type III responses. Therefore, evaluation of density‐dependent effects from conspecific individuals on size‐dependent predation may offer valuable information in understanding the factors involved in predator–prey interactions.

Omnivorous heteropteran predators are well known as high value naturally occurring natural enemies in several agroecosystems (i.e., Schaefer & Panizzi, 2000). Their important contribution to integrated pest management and biological control strategies is generally recognized, due to their predation efficiency and distribution in several habitats (Coll & Guershon, 2002; Fantinou et al., 2009; Molla et al., 2011; Perdikis et al., 2011; Pérez‐Hedo & Urbaneja, 2015). *Μacrolophus pygmaeus,* (Rambur) (Hemiptera Miridae) a generalist predator of whiteflies, aphids, mites, and several lepidopteran species including *Tuta absoluta* (Meyrick) (Lepidoptera Gelechiidae), is native to the Mediterranean area and is commonly used in pest management (Biondi et al., 2018; Chailleux et al., 2017; Perdikis & Lykouressis, 2000). Fantinou et al. (2008), Fantinou et al. (2009) showed that *Μ. pygmaeus* exhibited a type II functional response on each of the nymphal instars of *M. persicae*. Moreover, the presence of floral resources reduced the number of aphids consumed and therefore the functional response plateau (Maselou et al., 2014). Lampropoulos et al. (2013) reported a negative, antagonistic effect between two 5th‐instar nymphs of *M. pygmaeus* at intermediate but not at high levels of *Trialeurodes vaporariorum* (Westwood) (Hemiptera: Aleyrodidae) nymph's availability. However, foraging effort and hunting behavior of the predator were not strongly affected by the presence of another conspecific at intermediate densities of *Myzus persicae* nymphs (Sulzer) (Hemiptera: Aphididae) (Maselou et al., 2014). Michaelides et al. (2018) showed that interactive effects on consumption of eggs of *T. absoluta* of two 5th‐instar nymphs of *M. pygmaeus* were more intense at high prey densities. Adult body weight of predatory mirids was significantly reduced when nymphs raised in groups of 2, 4, 8, and 16 individuals, in the presence of prey, rather than reared in isolation (Arvaniti et al., 2019). Therefore, the above evidence supports that interference may exist between nymphs of *M. pygmaeus*.

In this study, we searched whether mutual interference interacts with predator body size. For this purpose, laboratory functional response experiments were conducted to test the effect of various predator–prey density combinations on feeding rate of similar sized individuals of either the 1st‐ or the 5th‐instar nymphs of *M. pygmaeus*. To achieve this, the effect of predator density, prey density, and predator body size on conspecific interference was explored by utilizing specialized functional response models to estimate parameters that show the trends of interference and its intensity among the variable traits tested.

## 2 MATERIALS AND METHODS

### 2.1 Insect culture

*Macrolophus pygmaeus* rearing was initiated from adults and nymphs collected from a tomato field in Co. Boeotia, central Greece (108 km NW of Athens, 23°18′29.46″E, 38°35′28.91″N). The climate in this area is continental where the average mean monthly temperature is 16.74°C and the annual precipitation is 582 mm according to the Hellenic National Meteorological Service. Insects were reared on potted eggplants, *Solanum melongena* (cv Bonica), and provided with sufficient quantities of *Ephestia kuehniella* Zeller (Lepidoptera: Pyralidae) eggs and *Artemia* spp. cysts (Entofood™, Koppert B.V., The Netherlands).

### 2.2 Functional response data

The experimental setup consisted of Petri dishes (9 cm diameter, 1.5 cm height) with a mesh‐covered hole in the lid (3 cm diameter) to reduce the accumulation of humidity. A tomato leaflet (6.45 ± 0.06 cm in length and 3.34 ± 0.13 cm in width) was placed, abaxial surface up, on a layer of water‐moistened cotton wool on the bottom of each Petri dish. In all of the experiments, 1st‐ or 5th‐instar nymphs of *Μ. pygmaeus* were used, <24 hr of age. Therefore, we expect interference interactions to be symmetric, that is, it affects all individuals the same. The 1st‐instar nymphs were collected from stems with eggs and 5th‐instar nymphs were obtained from first‐instar nymphs that were transferred from wood‐framed rearing cages to cages with potted tomato plants with food at 25°C, 65 ± 5% RH, and 16‐hr light per day, and left to develop until the 5th instar. Then, 1st‐ or 5th‐instar nymphs were introduced to caged tomato plants and were deprived of prey for 24 hr prior the beginning of the experiments to exclude the influence of variable hunger levels. Single and multiple predator treatments were conducted under controlled conditions of 25°C, 65 ± 5% RH and 16‐hr light per day. One, two, three, and four predators from each size class were introduced into a dish with the tomato leaflet and the prey. As prey, eggs of *E. kuehniella* were used (EPHEScontrol^®^, Agrobio S.L.,). In each of 1st‐instar predator treatment, the prey densities were 1, 2, 4, 8, and 16 individuals for single predators, 2, 4, 8, 16, and 32 eggs for two predators, 3, 6, 12, 24, and 48 eggs for three predators, and 4, 8, 16, 32, and 64 eggs when predator density was four nymphs. Likewise, for 5th‐instar nymphs of *M. pygmaeus* the prey densities tested were 1, 2, 4, 8, 16, and 32 *E. kuehniella* eggs for single predators, 2, 4, 8, 16, 32, and 64 eggs for two predators, 3, 6, 12, 24, 48, and 96 eggs when predator density was three nymphs, and 4, 8, 16, 32, 64, and 128 eggs when predator density was four nymphs. The five tested prey densities were adequate in order to test our hypothesis for 1st instars; however in the case of 5th instars, an additional prey density was included. In all cases, the prey/predator ratio was kept unchanged among the treatments. After 12 hr, the predators were removed from the dishes and the number of eggs consumed was recorded. There were performed 10–16 replicates at each prey density.

### 2.3 Statistical analyses

As the dependent variable is binomial (each egg prey was eaten or not at the end of the experiments), a logistic regression analysis on the proportion of egg prey eaten as a function of the initial egg density was conducted to determine the shape of the functional response (Trexler et al., 1988). A polynomial function (Juliano, 2001) was fitted using the method of maximum likelihood:

$\frac{N_{e}}{N_{0}}=\frac{\exp(P_{0}+P_{1}N_{0}+P_{2}N_{0}^{2}+P_{3}N_{0}^{3})}{1+\exp(P_{0}+P_{1}N_{0}+P_{2}N_{0}^{2}+P_{3}N_{0}^{3})}$, where *N_e_* denotes the total number of eggs eaten, *N*_0_ the initial egg number available, and *P*_0_, *P*_1_, *P*_2_, and *P*_3_ are the intercept, linear, quadratic, and cubic coefficients. If the linear coefficient is significantly negative, the proportion of egg prey eaten declines monotonically, suggesting a type II functional response; a significantly positive linear coefficient followed by a significantly negative quadratic coefficient suggesting a type III functional response (Juliano, 2001).

In all treatments, the linear coefficient was significantly negative (Tables 1, 2). Therefore, as the data indicated a type II functional response, the Crowley–Martin model (Crowley & Martin, 1989) was used to fit the data:

$$\frac{\mathrm{dN}}{\mathrm{dt}}=‐\frac{\mathrm{aNP}}{1+aT_{h}N+bt_{w}\left(P‐1\right)+aT_{h}bt_{w}N\left(P‐1\right)},$$

where *N* denotes the prey density; *P* the predator density; *a* the predator's attack rate, that is, the per capita prey mortality at low prey densities; *T_h_* the handling time which reflects the time, a predator spends on pursuing, subduing, eating, and digesting its prey; *b* the rate of encounter of a single predator with other predators; and *t_w_* the time wasted on an encounter. Although there are several models that allow for interference competition, we choose the Crowley–Martin model as in our recent study (Papanikolaou et al., 2016) it fitted several functional response data slightly better compared to the Beddington–DeAngelis (Beddington, 1975; DeAngelis et al., 1975), Holling type II (Holling, 1959b) and Hassell–Varley (Hassell & Varley, 1969) models. The parameters *b* and *t_w_* are structurally nonidentifiable since they always appear as a product. Therefore, we grouped them into one parameter *c* which is a positive constant describing the magnitude of interference among predators (Kratina et al., 2009; Papanikolaou et al., 2016; Skalski & Gilliam, 2001). Hence, the Crowley–Martin model become:

$$\frac{\mathrm{dN}}{\mathrm{dt}}=‐\frac{\mathrm{aNP}}{1+aT_{h}N+c\left(P‐1\right)+aT_{h}cN\left(P‐1\right)}.$$

**TABLE 1:** Estimated parameters (±*SE*) from the logistic regression analysis for *Ephestia kuhniella* eggs consumed by *Macrolophus pygmaeus* first‐instar nymphs

Parameter	*P* = 1	*P* = 2	*P* = 3	*P* = 4
Intercept	0.146 ± 0.175	0.663 ± 0.155	0.732 ± 0.139	0.828 ± 0.113
Linear	−8.784 ± 1.397 *	−8.472 ± 1.059 *	−9.265 ± 0.958 *	−9.827 ± 0.785 *
Quadratic	4.585 ± 1.404	1.995 ± 1.043	2.765 ± 0.935	3.270 ± 0.792
Cubic	−3.672 ± 1.353	−0.182 ± 0.999	−1.561 ± 0.853	−1.323 ± 0.710

Note

*P* denotes the predator density.

* Significant at *p* < .001.

**TABLE 2:** Estimated parameters (mean, 95% credible intervals) of the Crowley–Martin model, fitted to *Macrolophus pygmaeus* first‐instar nymph functional response data

	*a* (h^−1^)	*T_h_* (h)	*c*
*P* = 1	0.177	3.465	—
	(0.107–0.285)	(2.148–4.937)	—
*P* = 2	0.143	2.041	0.323
	(0.111–0.183)	(1.336–2.815)	(0.011–0.877)
*P* = 3	0.100	2.123	0.160
	(0.077–0.125)	(1.378–2.911)	(0.009–0.433)
*P* = 4	0.071	2.416	0.069
	(0.057–0.086)	(1.718–3.084)	(0.003–0.188)

Note

*P* denotes the predator density.

In case of a single predator (*P* = 1), the Crowley–Martin model is equal to the disk equation. Fitting was performed using the Bayesian approach suggested by Papanikolaou et al. (2016). In this task, a prior distribution is assigned to the parameters to express our prior beliefs about them before seeing the data. This information is then updated in the light of experimental data using Bayes theorem by multiplying it with the likelihood leading to the posterior distribution which contains all the information about the parameters. We used vague priors for most of the model parameters; typically, exponential distributions with very high variance (e.g., 1,000,000) to reflect our ignorance about them and allowed the posterior distribution of the parameters to be mostly informed by the data. However, we used the posterior distribution of the attack rate parameter, obtained from fitting the Holling model and by approximating it with a Gamma distribution (using the method of moments) as an informative prior, in order to overcome issues of practical nonidentifiability. Our inference procedure was based upon Markov chain Monte Carlo methodology. In particular, we employed a random‐walk Metropolis algorithm to draw samples from the posterior distribution of the parameters. The variance of the proposal distribution was tuned in order to achieve an acceptance rate of 25% (Roberts et al., 1997). All statistical analyses were conducted in R (R Core Team, 2019).

## 3 RESULTS

**FIGURE 1:**
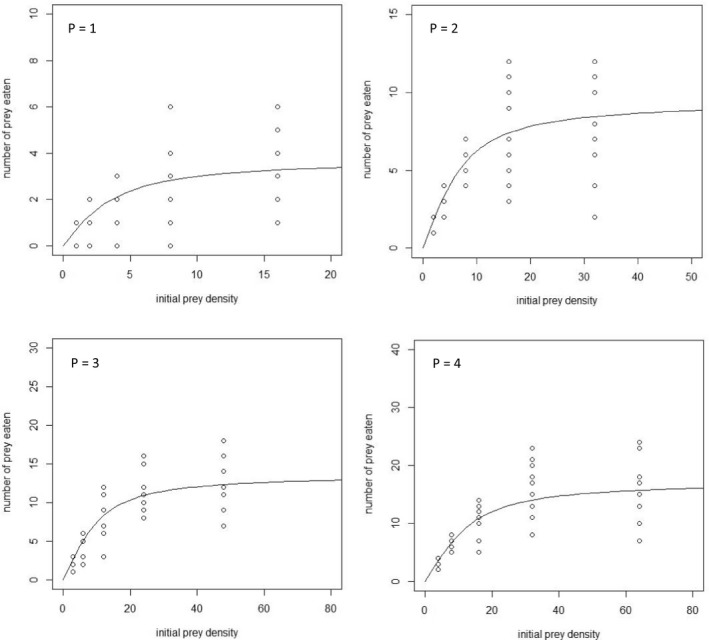
Fitting of the Crowley–Martin model to *Macrolophus pygmaeus* 1st‐instar nymph functional response data. *P* denotes the predator density

*Macrolophus pygmaeus* first‐instar nymphs exhibited type II functional response, as the linear coefficient of the polynomial function was significant negative in all predator combinations (Table 1). The Crowley–Martin model fitted the observed data reasonably well, as the fitted probabilities of the number of eggs consumed lie within the main bulk of the data (Figure 1). The estimated parameters for each predator combination, as well as the corresponding credible intervals, are presented in Table 2 and the corresponded posterior distributions in Figures S1–S4. When a single predator was used, the mean attack rate was 0.177 hr^−1^. As predator density increased, there was not statistically significant difference to the estimated attack rates when two or three predators were placed in the disk (0.143 and 1.000 hr^−1^, respectively). However, at the predator density of four nymphs, attack rate was significantly lower (0.071 hr^−1^) compared to single predator treatment. In addition, the estimated handling time for the single predator treatment was 3.465 hr, which did not differ significantly when two, three, and four predators were used (2.041, 2.123, and 2.416 hr, respectively).

**FIGURE 2:**
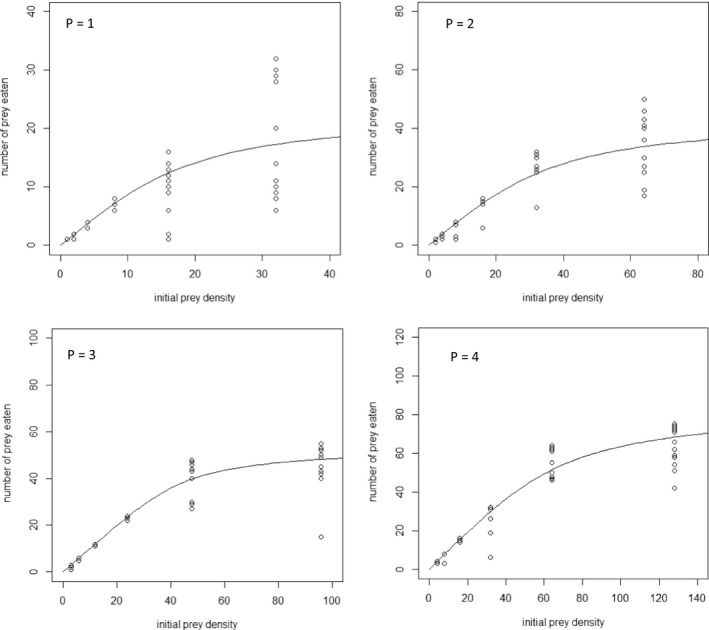
Fitting of the Crowley–Martin model to *Macrolophus pygmaeus* 5th‐instar nymph functional response data. *P* denotes the predator density

In case of fifth‐instar nymphs, the data indicated anew a type II functional response for all the experiments (Table 3) and the Crowley–Martin model fitted the data well (Figure 2). The estimated parameters of the model are presented in Table 4 and the corresponded posterior distributions in Figures S5–S8. The estimated mean attack rate for the single predator treatment was 0.283 hr^−1^. When two, three, and four predators were used, the mean attack rates were significantly lower compared to single predator (0.151, 0.188, and 0.094 hr^−1^, respectively). However, the mean handling times did not differ between predator treatments. When one, two, three, and four predators were used, the mean handling times were 0.542, 0.507, 0.605, and 0.534 hr, respectively.

**TABLE 3:** Estimated parameters (±*SE*) from the logistic regression analysis for *Ephestia kuhniella* eggs consumed by *Macrolophus pygmaeus* fifth‐instar nymphs

Parameter	*P* = 1	*P* = 2	*P* = 3	*P* = 4
Intercept	2.355 ± 0.300	1.636 ± 0.135	2.633 ± 0.225	2.493 ± 0.193
Linear	−10.825 ± 2.152*	−6.811 ± 0.968*	−10.763 ± 1.225*	−11.070 ± 1.304*
Quadratic	1.950 ± 2.055	−1.758 ± 0.982	−4.006 ± 1.317	1.326 ± 1.192
Cubic	5.432 ± 2.079	1.997 ± 1.034	6.808 ± 1.819	−3.261 ± 0.948

Note

*P* denotes the predator density.

* Significant at *p* < .001.

**TABLE 4:** Estimated parameters (mean, 95% credible intervals) of the Crowley–Martin model, fitted to *Macrolophus pygmaeus* fifth‐instar nymph functional response data

	*a* (h^−1^)	*T_h_* (h)	*c*
*P* = 1	0.283	0.542	—
	(0.224–0.347)	(0.419–0.674)	—
*P* = 2	0.151	0.507	0.115
	(0.131–0.173)	(0.406–0.615)	(0.007–0.320)
*P* = 3	0.188	0.605	0.076
	(0.160–0.218)	(0.481–0.723)	(0.005–0.212)
*P* = 4	0.094	0.534	0.034
	(0.085–0.104)	(0.452–0.617)	(0.002–0.084)

Note

*P* denotes the predator density.

The magnitude of interference (parameter c) among predators did not include zero values in 95% credible intervals in all cases. This further indicates that predation of *Macrolophus pygmaeus* follows the predator‐dependent assumptions of the Crowley–Martin model.

## 4 DISCUSSION

Our results showed density dependence of the functional response of *M. pygmaeus*. Density increase of the predator demonstrated a decrease in the estimated attack rate, and therefore the per capita searching predator efficiency, which indicates a limitation of its predation ability at low prey densities. An aspect that has received considerable attention in functional response studies is the limit to the number of prey attacked at high prey densities. It has been considered that handling time is the limiting factor for parasitoids and satiation for predators (Getz & Mills, 1996; Jeschke et al., 2002). Digestion and handling prey are discrete biological processes, as digestion influences the predators hunger level and its probability of searching for prey (Jeschke et al., 2002; Papanikolaou et al., 2014). It is likely that *M. pygmaeus* is a digestion‐limited predator, that is, digestion limits its predation efficiency. Thus, at low prey densities *M. pygmaeus* is not satiated, so that digestion breaks do not exist, and predators constantly search for prey. Therefore, we anticipate that at these densities, frequent encounters among predators distract individuals from prey searching activity. In contrast, at high prey densities the predator searching time is relatively low, and consequently the time loss due to encounters is much reduced. In addition, as individuals become satiated at these high prey densities, the time lost during digestion breaks may partially or fully accommodate the cost of interference; if digestion breaks proceed during interference interactions, the time cost of interference may be negligible (van Gils & Piersma, 2004).

Our data showed that *M. pygmaeus* first‐instar nymphs were less susceptible to interference competition than 5th instars as shown from the comparable values of attack rate to that of individual foragers. In the case of fifth‐instar nymphs, interference was evident since the values of attack rate at all foraging densities were significantly lower than that for single predators, and furthermore, significantly reduced with the increase of predator density. Thereafter, these results indicate a much stronger mutual interference among foragers of the fifth than the first nymphal instar. These results support the hypothesis that increase of predator size intensifies the phenomena of mutual interference among conspecific predators. Larger insect nymphs tend to show higher mobility (Hagstrum & Subramanyam, 2010), increasing the probability for encounter with competitors, so that they may be disoriented by the foraging activity.

In the outcome of mutual interference, besides predator density and size, arena/prey patch size may be a key component determining the intensity of interactions (Papanikolaou et al., 2016). We expect that the encounter rate among conspecifics is reversely associated with the patch size (Dostalkova et al., 2002; Kindlmann & Dixon, 2001). Therefore, a predator density threshold may exist above which conspecific interactions become significant for a given patch size. This hypothesis is supported by comparing our results in experiments conducted in same size arenas for both instar nymphs. Therefore, a predator dependence effect was recorded only at the highest density of 1st‐instar nymphs (i.e., 4 individuals), but in contrast, this effect emerged already when two 5th‐instar nymphs were enclosed together. In a previous study, the feeding rate of a predatory coccinellid was modified at low prey densities but only above a critical predator density (Papanikolaou et al., 2016).

A critical aspect of the evaluation of biological control agents is their response to increasing prey density. Most studies are focused on individual's functional response, which do not account for competition between individuals (e.g., Cabral et al., 2009; Jalali et al., 2010; Lee & Kang, 2004; Papanikolaou et al., 2011). In fact, the application of predator‐dependent functional response models may offer valuable outcomes, however is underutilized so far in the ecological literature, partly due to their complexity. Fitting such models to experiments can be challenging due to issues of practical identifiability, which arise when the experimental data do not provide enough information about the parameters of interest (Papanikolaou et al., 2016). The Crowley–Martin model shows statistically significant improvement over the purely prey‐dependent Holling's disk equation, as the 95% credible intervals of the parameter that account for interference competition did not include zero. This is in accordance with Skalski and Gilliam (2001), as they suggest the use of predator‐dependent functional response models over the Holling's disk equation. Their wider use in predator–prey interactions will help toward a better understanding of intraspecific interactions and its consequences on the stability and structure of food webs. In this task, our study shows that mutual interference is dependent on predator body size, with further impact on the attack rate. We expect this size dependence of the searching efficiency of foraging predators to affect predator–prey dynamics (e.g., Aljetlawi et al., 2004; De Roos et al., 2003; Hassell & May, 1973). The differences in competitive ability between different sized predators and the importance of the attack rate on stabilizing predator–prey dynamics have been highlighted by Persson et al. (1998), as it may result in different types of dynamics (stable, chaotic, etc.). Therefore, the quantitative assessment of mutual interference in predator–prey systems could lead to a further understanding of their dynamics.

The results of the current work deliver useful insights to be considered in the mass rearing of predators such as *M. pygmaeus* and their use in biological control. Firstly, prey densities should be kept at high levels particularly for larger nymphs rearing; otherwise, competition is increased reducing prey consumption rates with possible negative effects in the development and reproduction of the predator. Although much smaller in size than 5th‐instar nymphs, first‐instar nymphs’ predation rates are negatively affected by conspecific interactions, too.

Apart from prey risk reduction, intraspecific competition can lead predator individuals to prey switching by using other food resources for which less competition exists for their use (Svanbäck & Bolnick, 2007). Considering that optimal foraging theory supposes that predator may add alternative prey to its diet as a result of prey depletion (Stephens & Krebs, 1986), we expect that mutual interference can drive predatory mirids to alternative diets. According to our results, this should occur at low prey densities, and its intensity should increase with predator density, as a result of the competitive interactions among individuals. In such situations, phenotypic variation is likely to force individuals of a cohort to alternative food resources (Svanbäck & Bolnick, 2007). Searching these effects in case of omnivorous predators, in addition to alternative prey, alternative plant food resources must also be considered, as they may alter intraspecific interactions (Maselou et al., 2015).

In conclusion, our study highlights the importance of mutual interference on the predator functional response of a predatory omnivorous predator. It also provides evidence that predator size regulates mutual interference. These findings indicate that studying intraspecific interactions needs to utilize functional response modeling and take realistic levels of predator dependence into account. Future studies should investigate these effects under more complex environments, for example, field resembling conditions, or study the intensity of these effects in the presence of alternative food resources.

## CONFLICT OF INTEREST

The authors declare no conflicts of interest.

## AUTHOR CONTRIBUTION

**Nikos E. Papanikolaou:** Conceptualization (lead); Data curation (equal); Formal analysis (equal); Investigation (equal); Methodology (equal); Software (equal); Validation (lead); Writing‐original draft (equal). **Sofia Dervisoglou:** Data curation (equal); Investigation (equal). **Argyro Fantinou:** Conceptualization (equal); Methodology (equal); Writing‐original draft (equal). **Theodore Kypraios:** Formal analysis (equal); Software (equal). **Valmari Giakoumaki:** Investigation (equal). **Dionysios Perdikis:** Conceptualization (equal); Methodology (equal); Validation (equal); Writing‐original draft (equal).

## Supporting information

Appendix S1

## Data Availability

Data from the experiments are available on Dryad: https://doi.org/10.5061/dryad.2ngf1vhmj
