# Supplementary material for: Predator size affects the intensity of mutual interference in a predatory mirid
Source: Ecol Evol. 2020 Dec 29;11(3):1342–51. doi: 10.1002/ece3.7137 (PMC7863380; doi:10.1002/ece3.7137)

**APPENDIX 1**

**FIGURE A1**

Posterior distributions of the attack rate and handling time when the Crowley-Martin model fitted to the *Maclolophus* *pygmaeus* 1^st^ instar nymph single predator functional response data.


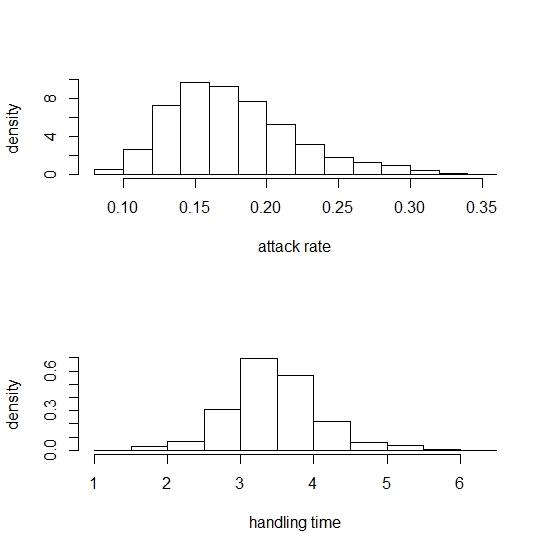


**FIGURE A2**

Posterior distributions of the attack rate, handling time and magnitude of interference when the Crowley-Martin model fitted to the *Maclolophus* *pygmaeus* 1^st^ instar nymph functional response data. Predator density was two nymphs.


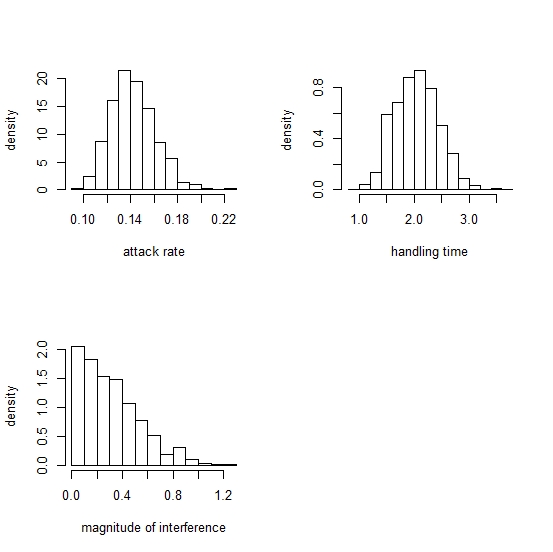


**FIGURE A3**

Posterior distributions of the attack rate, handling time and magnitude of interference when the Crowley-Martin model fitted to the *Maclolophus* *pygmaeus* 1^st^ instar nymph functional response data. Predator density was three nymphs.


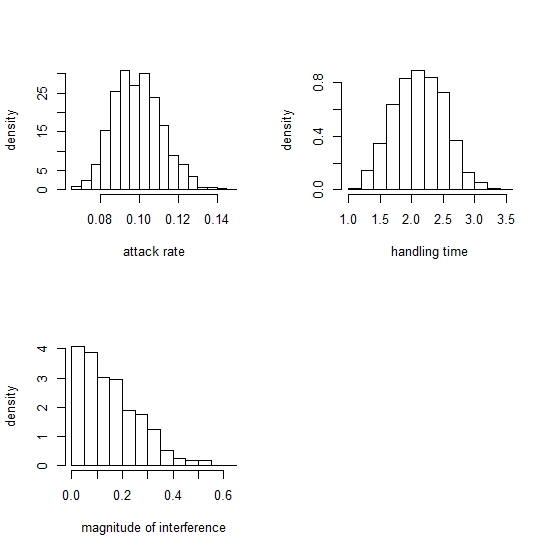


**FIGURE A4**

Posterior distributions of the attack rate, handling time and magnitude of interference when the Crowley-Martin model fitted to the *Maclolophus* *pygmaeus* 1^st^ instar nymph functional response data. Predator density was four nymphs.


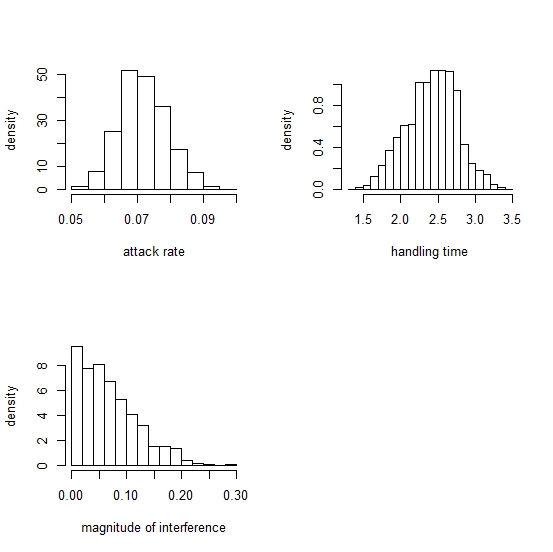


**FIGURE A5**

Posterior distributions of the attack rate and handling time when the Crowley-Martin model fitted to the *Maclolophus* *pygmaeus* 5^th^ instar nymph single predator functional response data.


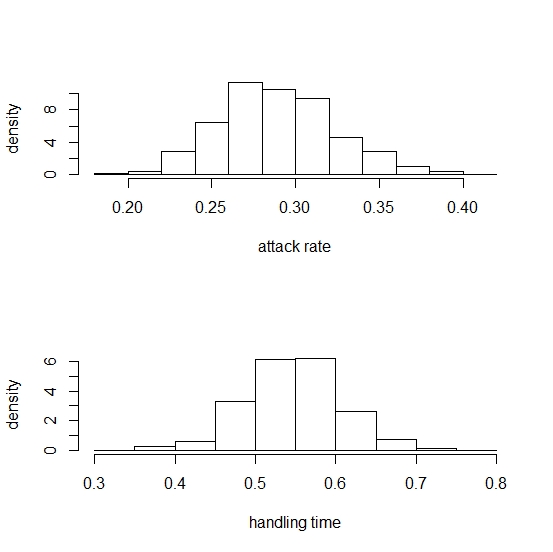


**FIGURE A6**

Posterior distributions of the attack rate, handling time and magnitude of interference when the Crowley-Martin model fitted to the *Maclolophus* *pygmaeus* 5^th^ instar nymph functional response data. Predator density was two nymphs.


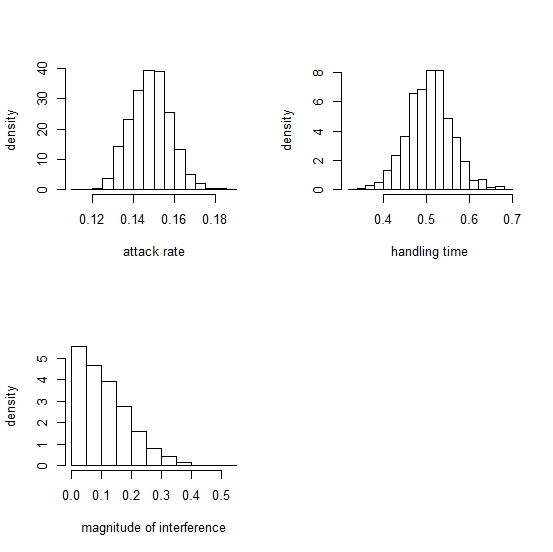


**FIGURE A7**

Posterior distributions of the attack rate, handling time and magnitude of interference when the Crowley-Martin model fitted to the *Maclolophus* *pygmaeus* 5^th^ instar nymph functional response data. Predator density was three nymphs.


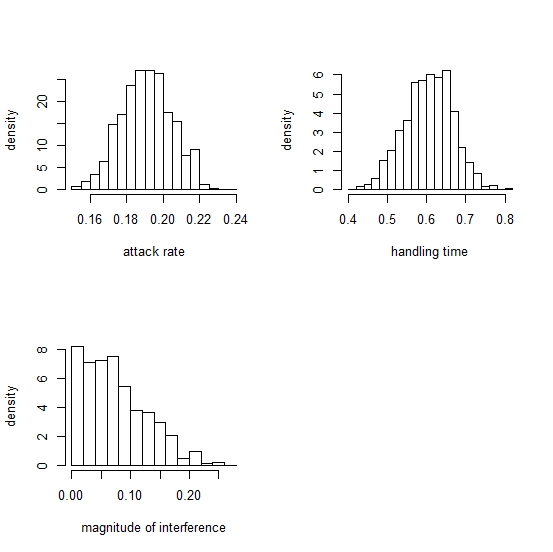


**FIGURE A8**

Posterior distributions of the attack rate, handling time and magnitude of interference when the Crowley-Martin model fitted to the *Maclolophus* *pygmaeus* 5^th^ instar nymph functional response data. Predator density was four nymphs.


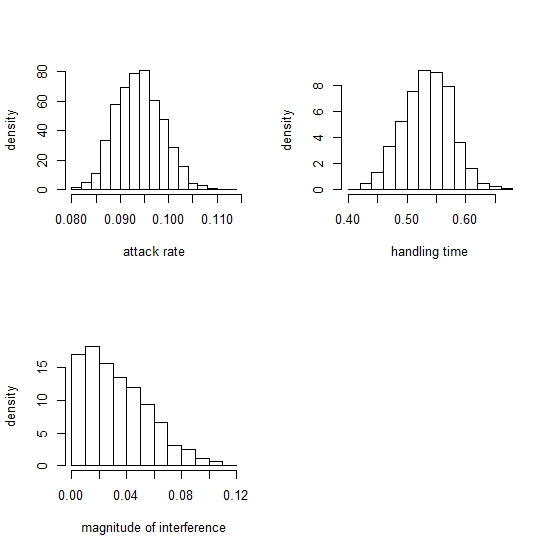

Supplement: Supplementary file 1 — Appendix S1 [file ECE3-11-1342-s001.docx]
